# An Address on Anæsthetics

**Published:** 1889-12

**Authors:** Dudley W. Buxton


ARTICLE V.

### AN ADDRESS ON ANÆSTHETICS.

BY DUDLEY W. BUXTON, M. D., C. S.

[Delivered before the Students' Society, London School of Dental Surgery.]

After some general remarks, Dr. Dudley Buxton said there were some points upon which it might not be uninteresting to enlarge.

In reference to the preparation of a patient preliminary to operation, it was in the case of nitrous oxide gas necessary to ensure that the patient was able to make full use of his breathing capacity. Simply loosening the upper portion of the dress is insufficient, although a wise procedure, since when the head is fully extended upon the trunk the neck is apt to become constricted by bands, collars, etc. As a rule it is easy to detect by the shape of the chest whether unduly tight clothing is worn, and if there be any suspicion of such a state of things a simple and expeditious test should be applied. The hands should be placed lightly upon the neck just below the collar bones and the patient instructed to take a deep breath. The hands should then be placed lightly upon the sides, just above the hips, and the patient again instructed to breathe deeply. If the corsets are too tight the amount of movement will be very slight, and then it is unnecessary to go through all this in every case, and here, as elsewhere, the administrator of the anæsthetic must rely upon his common sense and judgment. The necessity for attention to these preliminaries has been brought forcibly home to us all by the recent fatality in Edinburgh where a lady, aged 71, was so tightly laced that when under the influence of nitrous oxide gas she ceased to breathe, and as she was the subject of extensive heart disease, her life was lost through cardiac failure, consequent, I have no doubt, to the hampering of her breathing induced by tight laces and a distended stomach.

When these preliminaries have been adjusted it is important to see that the apparatus is most favorable as regards the patient's safety and comfort. I prefer Clover's methods to any I have yet seen, and always employ them. I think one cannot insist too strongly upon the necessity for an effectual expiration valve. The importance of this is determined by the necessity there is to abolish any asphyxial phenomena from nitrous oxide narcosis. Rebreathing exhaled air, or gas means taking back into the lungs and pulmonary circulation the poisonous refuse exhaled from the epithelium of the respiratory tract. Careful experience also has shown that even thoroughly purified air, which has once been breathed, is deleterious when rebreathed, and is, moreover, incapable of supporting life. This, then, is a further, and an additional reason why once breathed gas should be got rid of by a free expiration valve and not rebreathed. If what I have said is understood, it will be readily seen why I have so strong an objection to supplemental bags, and have so persistently decried their use.

Another important part of the apparatus consists of the gags, or as I prefer to call them "props." The mechanical gags, such as Buck's, are theoretically good, but are dangerous in practice from their liability to break, besides they get tooth indented and look dirty. In dental practice not only must all appliances *be* scrupulously clean but they must *look* clean. More than one almost fatal and even fatal result has followed breaking of spring gags.

During the administration it is not only essential that the anæsthetist should fulfil his duties efficiently, but it is as important for the patient to carry out the anæsthetist's instructions. Experience has taught me that it is far more efficacious to show a patient what to do, than simply to explain what you wish him to do. For instance, if you tell a patient to breathe deeply, he almost invariably takes a prolonged inspiration, and then holds his breath with the utmost pertinacity, and naturally complains, in the sequel, of feeling suffocated, since the narcosis is that mixed form not inappropriately called, "nitrous oxide and asphyxial narcosis." On the other hand, if you tell him to blow out with all his might, or as Mr. Cloves was in the habit of saying, "Blow as if you were blowing upon a looking-glass," he will obey and expire deeply, and in spite of himself will inspire just as deeply, for by voluntary effort we can hold our breath, but by no voluntary effort can we maintain the chest empty.

Upon the subject of the "mixed anæsthesia" of "nitrous oxide and asphyxia," I may say that I am surprised that any student of this hospital should make the statement that we do not know much about the physiological action of nitrous oxide gas! I venture to think that we know more about the physiological action of this agent than we do about any of the others, for the reason I am about to state. And again I join issue with Mr. Oliver when he tells us that we have learned nothing about the physiological action of the gas from the recent trials which have been made at our hospital of mixtures of oxygen and nitrous oxide. *Palmam qui meruit ferat*, let him wear the palm who has earned the prize. The great merit which belongs to my colleague Dr. Hewitt, lies in the painstaking and ingenious way in which he has striven to place in the range of practical anæsthetics the mixture of oxygen and nitrous oxide, but we must not forget that to one of the most original of French scientists of the past generation—M. Paul Bert, belongs the suggestion of employing nitrous oxide and oxygen, and the method is universally called after his name, Bert's method. To Bert we owe one great proof that nitrous oxide acts as a pure anæsthetic and not as an asphyxiant, for it was he who demonstrated that when given under pressure (true atmospheric pressure) with oxygen, the vital processes were carried on uninterruptedly even for over an hour of continuous inhalations. This took place ten years ago last July. Now as to the physiological action of nitrous oxide. Mr. Oliver does not appear to be aware that within the last ten years some rather elaborate and accurate researches have been undertaken which have shown, I think I may say, conclusively that—

1. Nitrous oxide acts as such, and that asphyxia does not in any way play a part in producing narcosis. Animals killed by asphyxiation evince evidence of suffering almost up to the last gasp, while those that die under nitrous oxide die absolutely insensient and unconscious. This fact has curiously enough been entirely overlooked by recent writers upon asphyxia.

2. Nitrous oxide possesses a specific action upon the cerebrospinal axis, producing well-marked vasomotorial changes leading to considerable increase in the bulk of the hemispheres and cord and developing interstitial pressure upon the cerebral and spinal tissues which determines narcosis. I may not however linger further, as to those who desire, can, by reference to the transactions of the Odontological Society for 1886 and '87, find the subject fully treated in two papers I contributed. Before leaving the subject of the mixture of oxygen and nitrous oxide, I must add a word of caution. We are not at present in a position to say how far it is safe to admit of continued inhalation of oxygen, and from the experience we have gained of its use in therapeutics, we are bound to admit it is not free from danger, owing to the excessive oxidation it gives rise to.

The completion of nitrous oxide anæsthesia is according to the reader of the paper, told by the loss of conjunctivol reflex. I think this sign is too uncertain to be deemed reliable, stertor and commencing jactitation, are, in my experience, the only truly reliable signs of unconsciousness.

But who are to be the administrators of nitrous oxide? It is unfortunately the fact that as the law at present stands, no one is bebarred from giving any anæsthetic, provided it is not done with an evil intent. Just as the law does not interdict any one from practising as a doctor or dentist, provided he does not assume a title which would induce the public to believe him to be a registered practitioner, so it does not discountenance any one from administering nitrous oxide. Of course, under various Acts the patient, or his friends could recover damages for injuries incurred through the maladministration of an anæsthetic, but it goes no further. However, there is very little doubt that at the present day it would go very hard with any one who had a death if the administrator were not a qualified or registered practitioner, as a qualification is presumptive evidence that the incumbent knows something about nitrous oxide administration.

Speaking of death and dangers of anæsthetics, I should like to draw attention to the importance of total inversion in the treatment of syncope occurring under an anæsthetic, and my remarks apply with equal force to cardiac failure under gas, and in the *first* or *second* stage of chloroform or ether narcosis. Nelaton was the first who insisted upon the value of inversion in chloroform faintness, and the method is called after his name. I have had some cases at the Dental Hospital which have strikingly exemplified the value of the manœuvre, cases in which faintness occurred, and alarming failure of the heart, and respiration stopped at the end of the operation and before any return no consciousness had taken place. I inverted, letting the head hang and raising the heels in the air, and at once the color returned and all faintness passed off. This manœuvre can be executed without any indelicacy or difficulty. In most of the dental chairs, arrangements exist by which the head can be lowered beneath the level of the heels, and this aids the process of inversion which may, or may not be completed by raising the heels according as the patient shows signs of recovery or not.

There has long been a mistaken idea in the minds of persons not conversant with the more modern and better methods of administering ether that persons take longer to become unconscious under ether than under chloroform, whereas the opposite is the case, the average time of etherization being in my hands two minutes, or when the gas is used as a preliminary one minute and a half, while chloroformization cannot safely be achieved in less than from five to eight minutes. I spoke just now of the value of inversion as a treatment for chloroform syncope, it is necessary therefore, that I should point out that discrimination in selecting the cases must be employed. In the first stage of chloroformization, so-called reflex syncope may occur, not only from fright, shock (imperfect anæsthesia) but also, as the experiments of Browne-Sequard, and others have shown, from the impinging of a too strong vapor upon the posterior pharyngeal wall, and so starting inhibiting action upon the heart through the pharyngeal plexus. It is in all these cases that inversion is most useful. When, however, syncope occurs as the result of cessation of respiration, when the lungs are engorged, and the right heart over-filled with blood, when, in short, asphyxia precedes heart failure, inversion is not to be practised, as it would only pour more blood into the already filled heart, and still further increase the difficulties of an organ already hampered.

There is much more I might say upon this subject, but as nitrous oxide is of greater interest to you than the alcohol series of anæsthetics, I have preferred to enlarge upon that, and cannot any longer monopolize your time and keep others from addressing you.—*Ohio Journal of Dental Science.*

